# Remission of Widespread Erythema Annulare Centrifugum After Empiric Oral Fluconazole in a 54-Year-Old Woman: A Case Report

**DOI:** 10.7759/cureus.118006

**Published:** 2026-10-16

**Authors:** Sehrish Bakhsh, Mohammad Almazied, Ohud Alghudifi, Fares Alammari

**Affiliations:** 1 Dermatology, Security Forces Hospital, Riyadh, SAU; 2 Dermatology, King Fahad Medical City, Riyadh, SAU; 3 College of Medicine, Almaarefa University, Riyadh, SAU

**Keywords:** antifungal therapy, delayed-type hypersensitivity, erythema annulare centrifugum, figurate erythema, fluconazole, gyrate erythema, reactive erythema, tinea incognito

## Abstract

Erythema annulare centrifugum (EAC) is a rare reactive dermatosis that can be difficult to manage in persistent cases. A 54-year-old woman presented with pruritic, annular, and polycyclic plaques that subsequently extended to the neck, trunk, upper back, hands, lower legs, and feet. A skin biopsy supported the diagnosis of superficial EAC on clinicopathologic correlation. After limited improvement with topical therapies and oral prednisolone, she received four weekly doses of empiric oral fluconazole 150 mg without adverse effects. The eruption improved, and approximately five months after treatment discontinuation, she remained clear apart from residual post-inflammatory hyperpigmentation. The temporal association suggests a possible treatment response; however, the absence of pretreatment mycologic testing and the possibility of spontaneous remission limit causal interpretation.

## Introduction

Erythema annulare centrifugum (EAC) is a rare figurate erythema. Lesions characteristically appear as annular, erythematous plaques that expand centrifugally with central clearing and frequently exhibit a trailing inner scale [1,2]. EAC is thought to be a delayed-type hypersensitivity reaction to diverse antigenic stimuli, with cutaneous fungal infections among the most commonly identified associated conditions in large clinicopathologic series [3-5]. Management is often difficult, especially in persistent cases, as standard anti-inflammatory therapies may yield only partial or temporary improvement. Empiric antifungal therapy has been suggested to address a possible occult fungal antigen source, but evidence in adults remains limited [6]. Here, we report a case of widespread, clinicopathologically supported EAC in an adult woman who achieved sustained remission after empiric oral fluconazole.

## Case presentation

A 54-year-old woman with a medical history remarkable for prediabetes treated with metformin presented with a one-month history of pruritic, hyperpigmented plaques over the bilateral antecubital fossae. At subsequent visits, the eruption had extended to the neck, trunk, upper back, hands, lower legs, and feet without associated pruritus at the newly involved sites. She had been managed elsewhere for a working diagnosis of psoriasis versus eczema. Oral loratadine, topical mometasone, paraffin-based emollients, topical betamethasone, and a calcipotriol-betamethasone combination were all tried without improvement. A three-week course of oral prednisolone produced only minimal benefit, and the eruption relapsed after it was stopped. The prednisolone dose could not be confirmed from the available records.

Cutaneous examination revealed well-defined, annular and polycyclic, hyperpigmented plaques with central clearing and a fine trailing scale at the inner border, consistent with the superficial variant of EAC, involving the neck, trunk, upper back, hands, lower legs, and feet (Figure 1).

**Figure 1:**
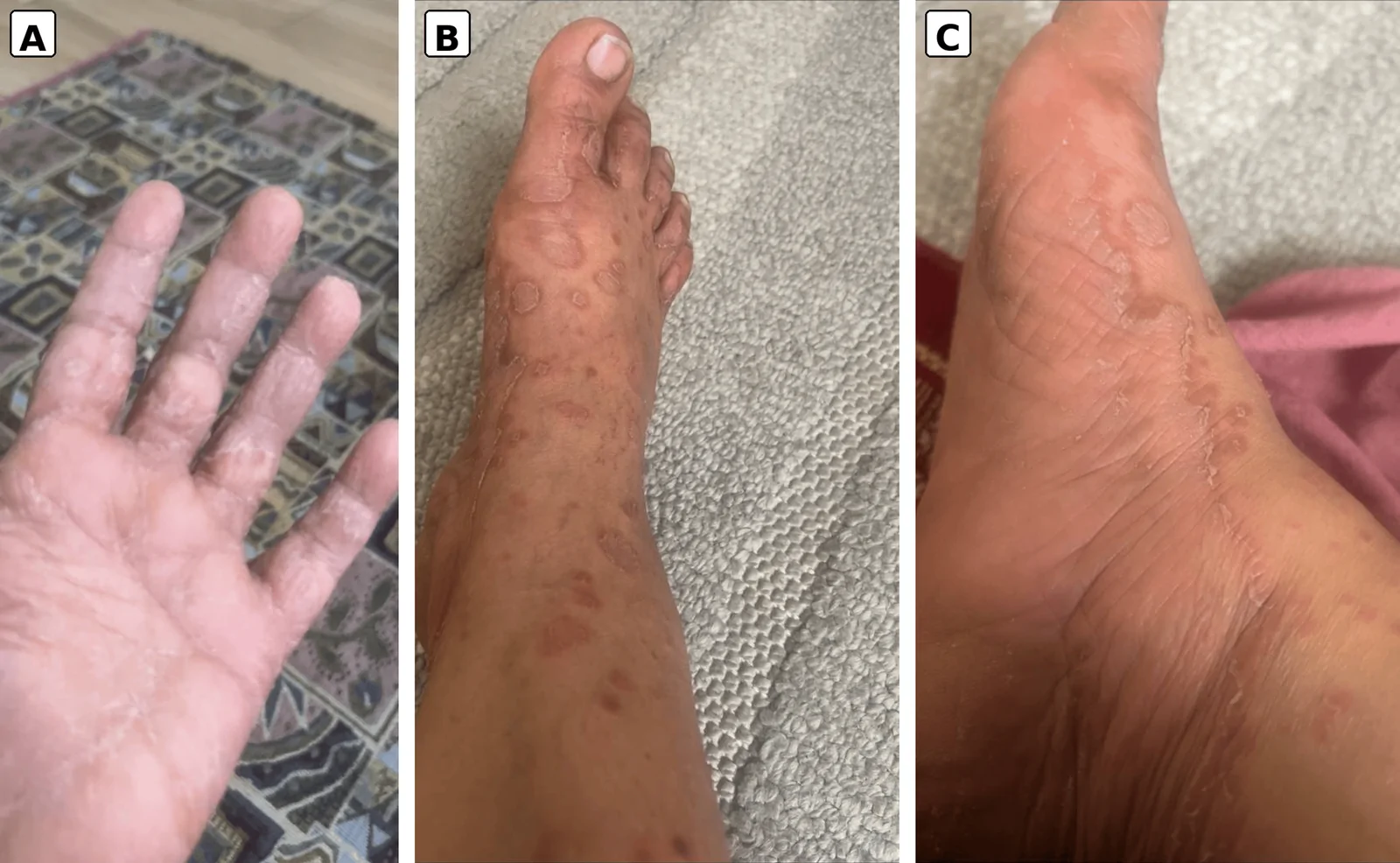
Clinical presentation at baseline (A) Well-defined, annular and polycyclic, hyperpigmented plaques with central clearing and a fine trailing scale at the inner border involving the hand. (B) Similar annular hyperpigmented plaques involving the dorsal aspect of the foot. (C) Similar morphology involving the medial and plantar aspects of the foot.

A 3 mm punch biopsy from a representative plaque on the right lower leg showed parakeratosis, acanthosis, spongiosis, a perivascular lymphocytic infiltrate, and focal interface lymphocytes, supporting the diagnosis of EAC on clinicopathologic correlation. The pathology report did not comment on fungal elements, and periodic acid-Schiff (PAS) and Grocott methenamine silver (GMS) stains were not performed and could not be done retrospectively on the archived tissue. Tinea corporis was the main alternative diagnosis, and after her earlier corticosteroid use, tinea incognito was a particular concern, given the annular, scaly plaques. Potassium hydroxide examination and fungal culture were not done before antifungal treatment was started. Laboratory evaluation, including complete blood count, liver function tests, lipid profile, thyroid-stimulating hormone, free thyroxine, rapid plasma reagin, treponemal antibody screen, HIV serology, hepatitis A IgM, hepatitis B surface antigen, and hepatitis C antibody, was unremarkable or negative. Metformin had been initiated after the onset of the eruption.

Empiric oral fluconazole 150 mg once weekly was initiated. During the fluconazole course, she also applied a topical mometasone-fusidic acid combination, twice daily for the first two weeks and once daily until the end of the four-week course. The patient first noticed improvement within two weeks. By the end of the four-week course, she estimated an 80% improvement in pruritus and hyperpigmentation across all involved sites. She completed all four weekly doses, and no adverse effects were reported at follow-up. Repeat laboratory tests after treatment, including liver function tests, were within normal limits. At the most recent follow-up, approximately five months after treatment discontinuation, she had no active inflammatory lesions or recurrence, only residual post-inflammatory hyperpigmentation, and had required no further treatment (Figure 2). 

**Figure 2:**
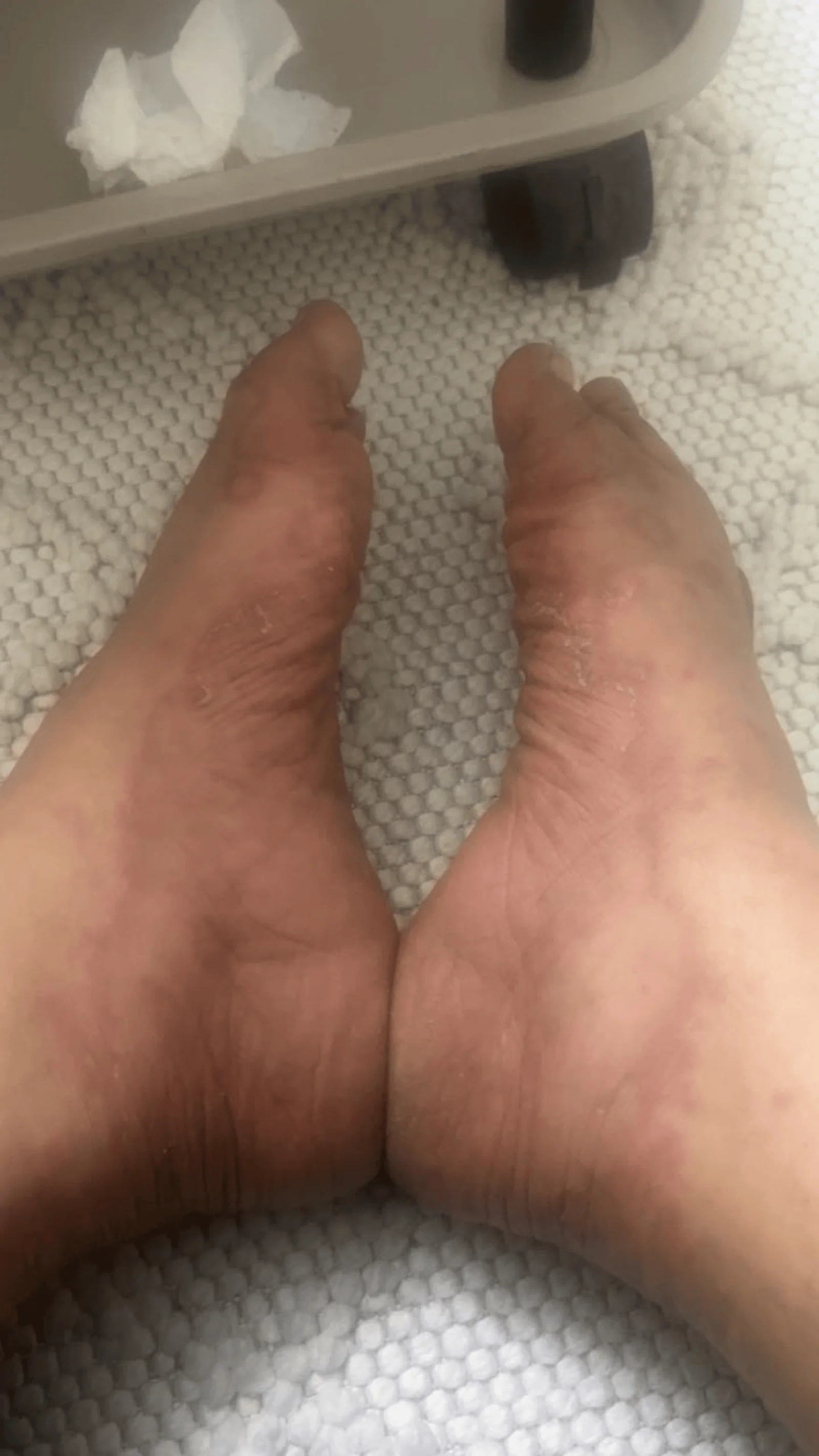
Clinical presentation at last follow-up Residual annular post-inflammatory hyperpigmentation involving the feet without new papules, plaques, scale, or other evidence of active inflammatory disease approximately five months after fluconazole discontinuation.

The clinical course is summarized in Table 1.

**Table 1:** Clinical timeline Summary of the patient’s clinical course from initial presentation through the most recent follow-up. EAC, erythema annulare centrifugum.

Time point	Clinical course
Initial presentation	One-month history of pruritic, hyperpigmented plaques over the bilateral antecubital fossae.
Subsequent visits	Extension to the neck, trunk, upper back, hands, lower legs, and feet; the newly involved sites were not pruritic.
Before fluconazole	No improvement with loratadine, topical mometasone, paraffin-based emollients, topical betamethasone, or calcipotriol-betamethasone. A three-week prednisolone course produced minimal benefit, followed by relapse after discontinuation; the dose was unavailable.
Diagnostic assessment	Examination showed annular and polycyclic hyperpigmented plaques with central clearing and trailing inner scale. A 3 mm punch biopsy from the right lower leg supported superficial EAC. Tinea corporis or tinea incognito was the principal alternative diagnosis; pretreatment potassium hydroxide examination and fungal culture were not performed.
Treatment week 0	Empiric oral fluconazole 150 mg once weekly was initiated, with topical mometasone-fusidic acid (twice daily for two weeks, then once daily until week 4).
Treatment week 2	The patient first noticed improvement.
Treatment week 4	The patient completed all four weekly doses without adverse effects and estimated an 80% improvement in pruritus and hyperpigmentation.
Approximately five months after discontinuation	No active inflammatory lesions, recurrence, or further treatment; residual post-inflammatory hyperpigmentation only.
Total disease duration	Approximately six months of active disease from onset to the start of fluconazole; approximately 12 months from onset to the most recent follow-up.

## Discussion

Erythema annulare centrifugum has a broad etiologic spectrum that includes infectious, neoplastic, autoimmune, and drug-related associations [1,5,7,8]. Of the infectious associations, cutaneous fungal infections are among the most commonly identified in large clinicopathologic series [3]. EAC has been proposed to be a delayed-type hypersensitivity reaction in which antigens from a remote focus may drive a secondary eruption [1,4]. It has also been reported as the first sign of an underlying lymphoproliferative malignancy, termed paraneoplastic EAC eruption [9]. Evaluation in adult patients should therefore be guided by history, examination, systemic symptoms, and age-appropriate screening [1,9].

Erythema annulare centrifugum management lacks a standardized approach because of its unpredictable course and limited treatment evidence [1]. Topical and systemic corticosteroids may provide only temporary relief, and lesions often return once treatment stops [6,10]. Calcipotriol, metronidazole, and azithromycin have been reported in isolated cases and a small observational series [11-13]. Remission has also been described in individual patients treated with etanercept, upadacitinib, and topical roflumilast, although these observations are insufficient to establish efficacy or a specific pathogenic pathway [10,14-16]. Kruse et al. reported a retrospective series of five pediatric patients with EAC of unknown cause who were treated empirically with oral fluconazole based on suspected occult yeast overgrowth. All patients showed improvement, three achieved complete clearance, and recurrences in two patients were treated with additional fluconazole [17].

This case contributes an adult observation to the limited literature on fluconazole use in EAC. The affected areas included the neck, trunk, upper back, antecubital fossae, hands, lower legs, and feet. Truncal involvement is commonly reported, whereas involvement of the hands and feet to this extent is less typical [7]. The decision to initiate empiric fluconazole was based on the reported association between EAC and cutaneous fungal infections [3], the clinical context of prediabetes, and the published pediatric fluconazole series [17]. Prediabetes was considered a contextual factor but did not establish the presence of occult fungal infection. Metformin was started after the eruption began, which makes it a less likely cause. The lesions relapsed when prednisolone was stopped but improved after a four-week course of fluconazole 150 mg once weekly, and there was no recurrence over approximately five months of follow-up. The temporal relationship and sustained remission are compatible with a treatment response, although spontaneous remission cannot be excluded.

One possible explanation is that fluconazole eliminated an unrecognized fungal antigen source that was maintaining the hypersensitivity reaction [1]. However, because pretreatment potassium hydroxide examination and fungal culture were not performed, tinea corporis or tinea incognito remains the principal alternative diagnosis and may have been treated directly by fluconazole. An isolated report has also described EAC temporally associated with topical and systemic azole exposure, with improvement after withdrawal and recurrence following re-exposure [18]. In the present case, the eruption improved rather than worsened during fluconazole therapy.

The most important limitation is that potassium hydroxide examination and fungal culture were not performed before fluconazole was started, so the fungal hypothesis rests on clinical reasoning rather than laboratory evidence. The 80% improvement is also the patient's own estimate, not a score on a validated scale. Concomitant topical mometasone-fusidic acid throughout the fluconazole course may have contributed to the improvement, although topical mometasone had previously been used without benefit. As a single case, the report cannot establish efficacy. Histopathology photomicrographs were unavailable for inclusion, limiting independent assessment of the microscopic findings. Tinea corporis or tinea incognito cannot be excluded, nor can spontaneous remission. The patient was referred for age-appropriate malignancy screening because of the reported paraneoplastic association of EAC but had not completed this evaluation at the time of writing [1,9]. Even so, the case documents sustained remission after empiric fluconazole in an adult, whereas published fluconazole data in EAC come mainly from children [17]. Future reports should include pretreatment fungal testing and a validated outcome measure.

## Conclusions

This case describes widespread, clinicopathologically supported superficial EAC followed by sustained remission after a four-week course of empiric oral fluconazole. The temporal association suggests that antifungal therapy may have contributed to improvement. Without pretreatment mycologic testing, however, a causal link cannot be established, and spontaneous remission remains possible.
